# *Heteroconium chaetospira* Induces Resistance to Clubroot via Upregulation of Host Genes Involved in Jasmonic Acid, Ethylene, and Auxin Biosynthesis

**DOI:** 10.1371/journal.pone.0094144

**Published:** 2014-04-08

**Authors:** Rachid Lahlali, Linda McGregor, Tao Song, Bruce D. Gossen, Kazuhiko Narisawa, Gary Peng

**Affiliations:** 1 Saskatoon Research Centre, Agriculture and Agri-Food Canada, Saskatoon, Saskatchewan, Canada; 2 College of Agriculture, Ibaraki University, Ibaraki, Japan; University of Nebraska-Lincoln, United States of America

## Abstract

An endophytic fungus, *Heteroconium chaetospira* isolate BC2HB1 (Hc), suppressed clubroot (*Plasmodiophora brassicae* -Pb) on canola in growth-cabinet trials. Confocal microscopy demonstrated that Hc penetrated canola roots and colonized cortical tissues. Based on qPCR analysis, the amount of Hc DNA found in canola roots at 14 days after treatment was negatively correlated (*r* = 0.92, *P*<0.001) with the severity of clubroot at 5 weeks after treatment at a low (2×10^5^ spores pot^−1^) but not high (2×10^5^ spores pot^−1^) dose of pathogen inoculum. Transcript levels of nine *B. napus* (*Bn*) genes in roots treated with Hc plus Pb, Pb alone and a nontreated control were analyzed using qPCR supplemented with biochemical analysis for the activity of phenylalanine ammonia lyases (PAL). These genes encode enzymes involved in several biosynthetic pathways related potentially to plant defence. Hc plus Pb increased the activity of PAL but not that of the other two genes (*BnCCR* and *BnOPCL*) involved also in phenylpropanoid biosynthesis, relative to Pb inoculation alone. In contrast, expression of several genes involved in the jasmonic acid (*BnOPR2*), ethylene (*BnACO*), auxin (*BnAAO1*), and PR-2 protein (*BnPR-2*) biosynthesis were upregulated by 63, 48, 3, and 3 fold, respectively, by Hc plus Pb over Pb alone. This indicates that these genes may be involved in inducing resistance in canola by Hc against clubroot. The upregulation of *BnAAO1* appears to be related to both pathogenesis of clubroot and induced defence mechanisms in canola roots. This is the first report on regulation of specific host genes involved in induced plant resistance by a non-mycorrhizal endophyte.

## Introduction

Clubroot disease, caused by *Plasmodiophora brassicae* Woronin, is a serious threat to canola (*Brassica napus* L.) production in western Canada [1], [2]. Fungicides applied as a soil drench may reduce disease impact on vegetable brassicas [3], [4], but this application method is impractical on canola [5]. Host resistance is currently the main strategy for clubroot management in canola, but sources of resistance are limited [6] and generally not durable [7]. Strategies that reduce infection may be used in combination with host resistance to bolster the performance and potentially longevity of resistant cultivars.

Several soil microbes have been shown to suppress clubroot in Brassica crops [5], [8], [9], [10]. *Heteroconium chaetospira* (Grove) M.B. Ellis is of particular interest because of its ability to colonize the roots of Chinese cabbage (*B. rapa* L. subsp *pekinese*) [11], [12] and suppress clubroot and Verticillium Yellows [13]. This species belongs to a loose group of dark-septate endophytes (DSE), largely anamorphs of ascomycetous fungi, which colonizes roots without causing visible symptoms [14], [15]. DSEs capable of colonizing roots of Brassica crop species are of particular interest because thioglucosides, found in roots of these plants, inhibit arbuscular mycorrhizae (AM) [16] and prevent AM-based symbiosis [17]. This symbiosis is generally beneficial to the plant host, and some AMs even induce resistance against diseases via activation of host-defence mechanisms [18], [19]. Root colonization by other endophytes in Chinese cabbage also benefit the host by promoting growth [20] and inducing resistance or tolerance to diseases [13], [21]. An isolate of *H. chaetospira* has been shown to colonize Chinese cabbage roots extensively [11], [12], where it obtains carbon from roots, supplies the host plant with nitrogen [22] and causes induced systemic resistance (ISR) to clubroot and leaf-spot diseases [13], [23]. This interaction may provide an on-going stimulus for ISR and a longer period of plant protection [24], [25] relative to synthetic fungicides that tend to break down rapidly after application [5].

An isolate of *H. chaetospira* (BC2HB1) was obtained from a Canadian forest soil using bait plants [26]. The fungal identification, based only on morphological traits, was considered tentative. It was also not known that if this isolate could colonize canola roots and protect the plant against clubroot. Additionally, mechanisms used by *H. chaetospira* in inducing host resistance against clubroot were not understood. When challenged with foliar pathogens, canola plants showed systemic acquired resistance (SAR) via activation of genes encoding several pathogenesis-related proteins (PRs) [27] through the salicylic acid (SA) biosynthetic pathways. In a preliminary study using microarray, however, the *H. chaetospira* isolate BC2HB1 did not activate genes involved in SA pathways, but upregulated several genes involved in the jasmonic-acid (JA) and ethylene (ET) pathways significantly [28].

The objectives of this study were to: i) verify the tentative designation of isolate BC2HB1 as *H. chaetospira* based on rDNA sequences, ii) determine and quantify the colonization of canola roots by BC2HB1, iii) assess the impact of root colonization by BC2HB1on development of *P. brassicae* in canola roots and on biocontrol of clubroot, and iv) analyze the activity of canola genes (*Bn*) involved in biosynthetic pathways related to plant defence induced by BC2HB1 treatments.

## Materials and Methods

### Molecular identification of BC2HB1

The endophytic fungal isolate BC2HB1 was isolated from a forest soil sample (200 g) collected in the Jackman Flats provincial park, British Columbia, Canada in 2004 [26]. No specific permission was required for the collection of a small soil sample at the location, and this activity did not involve any endangered or protected species. This fungus was considered an isolate of *Heteroconium chaetospira* based on morphological traits [26], but was a different isolate from that used on Chinese cabbage against clubroot previously [8]. Molecular characterization was conducted to confirm the identity of this specimen. A culture of BC2HB1was grown in liquid potato dextrose broth at 25°C, harvested by filtration through a 0.22-μm membrane (Millipore Corp, Billerica, MA), and its DNA was extracted using the DNeasy Plant Mini Kit (Qiagen, Montreal, QB) following the manufacturer's protocol. Internal transcribed spacer (ITS) regions of the fungus was amplified using a Veriti™ thermal cycler (Lift Technologies, Montreal, QC) and primers ITS4 (5′-TCCTCCGCT-TATTGATATGC-3′) and ITS5 (5′- TGGAAGT-AAAAGTCGTAACAAGG-3′) [29]. Amplification reactions were carried out in a volume of 50 μl using a BioLab Kit (BioLab, West Hill, ON) and manufacture's protocols in the following program: 5 min at 94°C, followed by 29 cycles of 30 s at 94°C, 30 s at 59°C and 1 min at 72°C, and finally 7 min at 72°C. PCR amplicons were purified using QIAquick PCR purification kit (Qiagen) following manufacturer's protocols prior to sequencing at Plant Biotechnology Institute, Saskatoon, SK. Sequences were compared with those deposited in the Genbank database using the nucleotide Blast program of NCBI.

### Biocontrol efficacy of BC2HB1against clubroot on canola

A solid-substrate fermentation process was used to produce a granular formulation of BC2HB1. Fifty grams of hull-less cracked barley (cv. Tercel) and 40 ml water were poured into a 500-ml wide-mouth Erlenmeyer flask. The flasks were covered with aluminum foil and autoclaved twice at 121°C for 30 min with a 24 hour stand time between each sterilization process. Fungal inoculum was prepared by growing isolate BC2HB1 on 50% oatmeal agar in Petri dishes for 4 weeks, then homogenizing each culture in a Waring blender with 150 ml of sterile water. Each flask was inoculated with 20 ml of the homogenate and incubated at 23°C for 4 weeks. The infested grain was dried in trays at 26°C and ground in a Wiley Mill (Thomas Scientific, Swedesboro, NJ) through a 0.5–1.0 mm screen. The ground inoculum was kept in a freezer at −20°C until required.

Tall, narrow plastic pots (3.5 cm×20 cm “conetainers”, Stuewe and Sons, Corvalis, OR) were filled with soil-less planting mix (pH 5.8–6.2, Sunshine #3, SunGro Horticulture, Vancouver, BC) and soaked twice to saturation with water adjusted to pH 6.3 using 2M HCL. The granular formulation of BC2HB1was mixed with planting mix at three rates (0, 1.25%, and 2.5%, w/v) and tested against two pathogen inoculum doses (2×10^5^ and 2×10^6^ spores pot^−1^) added at 0 or 7 days after seeding (DAS). The experiment was carried out using a 3×2×2 factorial arrangement [BC2HB1 rates ×*P. brassicae* (Pb) doses × inoculation timing] with a completely randomized design and a total of 12 treatments. Seven plants were included with each treatment in a trial repetition and this was consistent with the protocol used to assess the efficacy of two biofungicides against clubroot on canola [10], [36], [37]. A formulation blank was incorporated at 2.5% (w/v) into a planting mix and used for 0 BC2HB1 treatments. An autoclaved (121°C for 30 min) BC2HB1 formulation (2.5%) was used as an additional check and inoculated with Pb only at 7 DAS. The experiment was conducted three times, and all materials were prepared independently for each test.

The canola cv. Fortune RR was used as a susceptible host. After seeding in the BC2HB1 amended planting mix, resting-spore suspensions of Pb at 2×10^5^ and 2×10^6^ spores pot^−1^ were applied, respectively, as a soil drench at seeding (0 DAS) and at 7 DAS. Seeded pots were placed in a growth cabinet (18–23°C) with a 14-h photoperiod (512 μmoL m^−2^s^−1^) for five weeks before assessment of clubroot severity on individual plant using a standard 0–3 scale [30]. A disease severity index (DSI) was calculated over the 7 plants of each treatment in a trial repetition using the formula described previously by Lahlali et al. [36].

### Quantification of *H. chaetospira* (Hc) and Pb in canola roots using qPCR

In conjunction with each of the efficacy trial repetitions described above, three plants (additional to the 7 plants used for efficacy assessment) were removed at 14 DAS from each treatment for quantification of BC2HB1 and Pb in root tissues. By 14 DAS, the secondary infection by Pb has generally reached the peak [34] and significant gene induction by BC2HB1 can be detected in canola roots [28]. Root samples were washed in running tap water for 5 minutes, ground to a powder in liquid nitrogen (minimum 0.2 g pre replicate), and gnomic DNA (gDNA) samples were isolated using the Plant DNeasy kit (Qiagen). The amount of Hc and Pb DNA was analyzed separately using quantitative PCR (qPCR). Root tissues from a single plant were used as a biological replicate. The qPCR was performed in a reaction volume containing a 2-μL template DNA, 0.1 μl of each primer (50 nM), 5.28 μL SYBR Green I PCR Master Mix (ABI), and 7.98 μl of ultra-sterile deionized water. Amplification and quantification was performed using a standard StepOne™ qPCR Detection System (Life Technology).

For quantification of Hc DNA, following thermocycling conditions were used: An initial 5 min at 95°C, 50 cycles of 15 s at 95°C, 30 s at 58°C, and finally 30 s at 72°C. The threshold levels for signal detection were set automatically by the software and the quantification was done using a standard curve developed via a ten-fold dilution (2×10^0^ to 2×10^−4^ ng) of gDNA prepared from a pure BC2HB1 culture. The reactions were conducted using primers designed for this project by the Life Technology based on internal specific genes from 18S ribosomal RNA (Acc. No. DQ521604): F-HC2 (ACCTTGGACTTGGCTGATCTGT) and R-HC2 (AAGGAAAGACCCGACCGAAT). To quantify the amount of Pb gDNA, the DNA extracted from root samples in the above-mentioned step was tested using the primers Pb4-1 and PbITS6 [31], and the protocol described earlier by Lahlali et al. [10]. Three technical replicates (DNA samples) were used for each biological replicate in qPCR testing to establish standard regression curves between the threshold cycle (Ct) and the logarithm of the template concentration. A melting-curve analysis was performed for qPCR along with electrophoresis (2% gel) post amplification to ensure only the target PCR product had been amplified.

### Observation of canola root colonization by BC2HB1 using confocal microscopy

To confirm the root colonization by BC2HB1, a separate experiment was conducted using root samples from plants treated with the BC2HB1 formulation at 1.25% and 2.5% (w/v), respectively, and inoculated with Pb. A formulation blank (2.5%) and an autoclaved BC2HB1 (2.5%) were used as controls. These formulations were incorporated in planting mix, and a resting-spore suspension of Pb was pipetted to each pot after seeding at 2×10^5^ spores pot^−1^. At 14 DAS, three plants were removed from each treatment, washed with tap water repeatedly, cut into 1-cm-long pieces, and kept in 70% ethanol overnight.

For confocal microscopy, root samples were placed in micro-centrifuge tubes wrapped with aluminium foil to prevent light from entering. Each sample of about 10 root pieces was stained in AlexaFluor 633 (conjugated with 10 μg/ml Wheat Germ Agglutinin, Life Technologies, Burlington, ON) for 10 min in the dark, rinsed 10 times in phosphate-buffered saline, and then stained in 5-mM Syto 13 Green (Invitrogen, Burlington, ON) in Tris-EDTA buffer for 10 min in the dark, rinsed with sterile deionized water, and stored in a small volume of water at 4°C in the dark until use. The Wheat Germ Agglutinin conjugated to AlexaFluor 633 and Syto 13 Green fluorescent nucleic acid stain produced the best differential staining of Hc and Pb in canola root tissues among a range of dyes evaluated (R. Lahlali, unpublished data). Stained root pieces were mounted on a microscope slide and examined using a Zeiss LSM 710 confocal microscope (Carl Zeiss Canada Ltd. North York, ON). Images were acquired by excitation at 633 and 488 nm and emission with a long pass 640 and a band pass 493–604 nm filter, respectively, for the AlexaFluor 633 and Syto 13 Green dyes. Images were processed using the software Imaris (Bitplane USA, South Windsor, CT), and histogram stretching plus gamma adjustment were used to optimize images. This experiment was conducted twice.

### Activity of phenylalanine ammonia lyases (PAL)

In a separate experiment, leaf, stem and root samples were taken from canola plants treated with the BC2HB1 formulation (0, 1.25%, 2.5%) and inoculated with a resting-spore suspension of Pb at 2×10^5^ spores pot^−1^ as described earlier. There were 6 treatments for this analysis: negative control (formulation blank, no Pb), pathogen control (formulation blank + Pb), BC2HB1 alone at 1.25% and 2.5%, and BC2HB1 at 1.25% and 2.5% plus Pb, respectively. Samples were collected at 14 DAS, homogenized separately in 0.1 M sodium borate buffer (pH 8.8) at 4°C, the homogenates centrifuged at 12,000 *g* at 4°C for 20 minutes and the supernatant kept for the analysis of enzyme. An assay mixture consisted of 1.5 ml borate buffer (150 μM), 1.0 ml deionised water, 1 ml L-phenylalanine (Sigma–Aldrich Canada, Oakville, ON) solution (10 μM) and 0.5 ml the supernatant. After incubation at 38°C for 2 h, 50 μl of 5N HCL was added to the mixture to stop the reaction. The PAL activity was quantified by measuring the total amount of t-cinnamic acid formed in each sample using a SPECTRAmax Plus spectrophotometer (Molecular Devices, Sunnyvale, CA) at 290 nm relative to a reference PAL activity in controls without the addition of L- phenylalanine [32], [33]. A completely randomized design was used and the experiment was conducted twice, with 3 replicates per treatment in each repetition.

### Gene expression in canola roots treated with Hc and Pb

In a separate experiment, root samples were taken at 14 DAS, and transcript levels of nine genes that encode enzymes involved in several biosynthetic pathways potentially related to plant defence were analyzed using qPCR. Three treatments were applied: formulation blank (control), BC2HB1 formulation (2.5%) plus Pb (2×10^5^ spores pot^−1^) and Pb alone, with 4 replicates (plants) per treatment. The treatments were applied similarly as in the earlier experiments described above. Total RNA was isolated from fresh root tissues using the RNeasy Plant Mini Kit (Qiagen) following manufacturer's protocols. The RNA concentration was measured using the Nanodrop ND-1000 (Nanodrop Technologies, Wilmington, DE). First-strand cDNA was synthesized from 5 μg of total RNA using Super Script First Strand Synthesis (Life Technologies).

The expression of basic defence-related genes *BnPR-1*, *BnPR-2* and *BnPR-5* [27], and genes involved in signaling hormone (*BnSAM3, BnAAO1*, *BnACO* and *BnOPR2*) and phenylpropanoid (*BnCCR* and *BnOPCL*) pathways [35] were analyzed due to their potential connection to ISR and SAR against several diseases on canola [27], [35], [36], [37]. To determine the relative expression of these genes, primers were used with qPCR in the following steps: a 2-μl gDNA sample was brought to a total reaction volume of ≈20 μl with 10 μl of SYBR Green-I, 0.5 μl of each primer (50 nM), and 7 μl of DEPC-water [1 ml 0.1% diethylpyrocarbonate in 1 L deionized water). The qPCR amplification was carried out for each of the target genes using the primers and conditions reported previously [27], [35], and the actin gene was used as the housekeeping gene control for qPCR [35]. The transcript level of a target gene in each treatment relative to the control was measured using the qPCR based on the Ct for treated and control plants, respectively. Differences in the relative expression of each gene were determined based on the least significant difference (LSD, *P*≤0.05). Four biological replicates, each consisting of three technical replicates (RNA samples), were used per treatment and the experiment was conducted twice.

### Statistical analysis

Statistical analyses of data were carried out using the SAS software (version 9.1, SAS Institute, Cary, NC). A Log-based transformation was applied to DSI (%) for normal distribution of the data prior to analysis. The homogeneity of variances was checked using the Bartlett's Test for DSI, DNA quantification, PAL activity and gene expression data from repeated tests, and confirmed prior to pooling of data for analysis. Technical replicates used in qPCR were analyzed automatically by the StepOne™ software, and the printout of accepted means over a biological replicate was used for statistical analysis using SAS. Analysis of variance (ANOVA) was conducted using the Generalized Linear Model (PROC GLM) for data from all experiments. In ANOVA of DSI and DNA data, interactions between the BC2HB1 rate, pathogen dose and inoculation timing were insignificant, and treatment means therefore were compared using Fisher's Protected LSD (P<0.05). Only non-transformed values were presented in Results.

## Results

### Molecular identification of BC2HB1

Based on a Blast search of the sequence of the ITS regions of 5.8s rDNA, the isolate BC2HB1 showed a 97% similarity to an isolate of *Heteroconium chaetospira* (syn. *Cladophialophora chaetospira*) deposited in the GenBank under the accession number DQ521604. The sequence of BC2HB1 has been deposited in the GenBank under the accession number HQ871874.

### Efficacy of BC2HB1 against clubroot on canola

**Figure 1:**
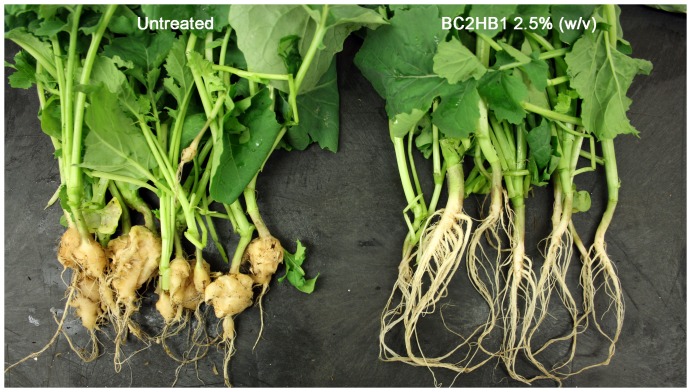
The plants on the right illustrate suppression of clubroot on canola by a granular formulation of *Heteroconium chaetospira* BC2HB1 incorporated into the planting mix at 2.5% (w/v). Plants on the left are from an untreated control. Each plant was inoculated with a suspension of *Plasmodiophora brassicae* resting spores at 2×10^5^ spores pot^−1^ immediately after seeding.

The *H. chaetospira* isolate BC2HB1 (Hc hereafter) applied at the 2.5% rate sometimes suppressed clubroot completely (Figure 1) at the low pathogen inoculum (2×10^5^ spores pot^−1^), but more often reduced the disease severity moderately (Table 1). On average, the Hc formulation at the 2.5% rate reduced DSI by 68% and 57%, respectively, against the low pathogen dose applied at 0 and 7 DAS (Table 1) relative to the formulation control. At the 1.25% rate, however, the treatment reduced DSI by 48% at the low pathogen inoculum applied at 0 DAS but not at 7 DAS. Neither Hc rate was effective at the high dose of pathogen inoculum (2×10^6^ spores pot^−1^). No clubroot symptoms were observed on non-inoculated control plants (data not shown).

**Table 1:** Clubroot severity (disease severity index - DSI) on canola plants treated with a granular formulation of *Heteroconium chaetospira* strain B2HB1 (Hc) under high and low inoculum pressure of *Plasmodiophora brassicae* (Pb) added to the growth medium at seeding (0 DAS) or 7 days after seeding (7 DAS) (n = 3)^x^

Hc treatment and rate (w/v, %)	Low Pb (2×10^5^ spores pot^−1^)		High Pb (2×10^6^ spores pot^−1^)	
	0 DAS	7 DAS	0 DAS	7 DAS
Control (0%)	56 (16.4) de^y^	42 (6.9) cde	53 (18.7) cde	44 (11.8) cde
Low Hc (1.25%)	29 (14.5) abc	30 (3.1) abcde	68 (11.5) e	41 (13.0) bcde
High Hc (2.5%)	18 (4.0) ab	18 (6.3) a	27 (10.6) abcd	26 (4.1) abcd
Autoclaved Hc (2.5%)	Not tested	30 (1.7) abcde	Not tested	28 (3.2) abcd

x DSI data from three trial repetitions were used as biological replicates in the analysis.

y Numbers in brackets are standard errors of the mean. Means followed by the same letter do not differ (protected LSD at *P*≤0.05).

### Quantification of Hc and Pb in canola roots using qPCR

gDNA samples extracted from the mycelia of HC showed a single PCR amplicon with about 65 bp (data not shown). At 14 DAS, Hc gDNA was detected in all root samples, with a strong positive correlation between the threshold values and the amount of DNA in roots (*r* = 0.99, *P*<0.001). The amount of Hc DNA in canola roots was not affected by the presence of Pb inoculum at 2×10^5^ spores pot^−1^ (Table 2). At the higher Hc rate (2.5%), the amount of Hc DNA appeared significantly higher with Pb inoculation than without. No Hc DNA was detected in any of the control root samples (data not shown).

**Table 2:** Colonization of canola roots by *Heteroconium chaetospira* strain BC2HB1 (Hc) based on qPCR, with or without inoculation with *Plasmodiophora brassicae* (n = 9).

Hc treatment and rate (w/v, %)	Hc DNA (ng g^−1^ fresh root)^x^	
	Without Pb	With Pb^y^
Low Hc (1.25%)	0.12±0.01 b^z^	0.24±0.05 b
High Hc (2.5%)	0.18±0.02 b	0.64±0.01 a
Autoclaved Hc (2.5%)	0.04±0.00 c	0.04±0.00 c

x Plants receiving no BC2HB1 were used as a negative control, and *H. chaetospira* DNA was not detected in roots of control plants.

y 2×10^5^ resting spores pot^−1^. The data for 0 and 7 DAS were pooled within each repetition.

z Data from repeated trials were pooled for analysis. Means followed by the same letter do not differ (protected LSD at *P*≤0.05).

**Figure 2:**
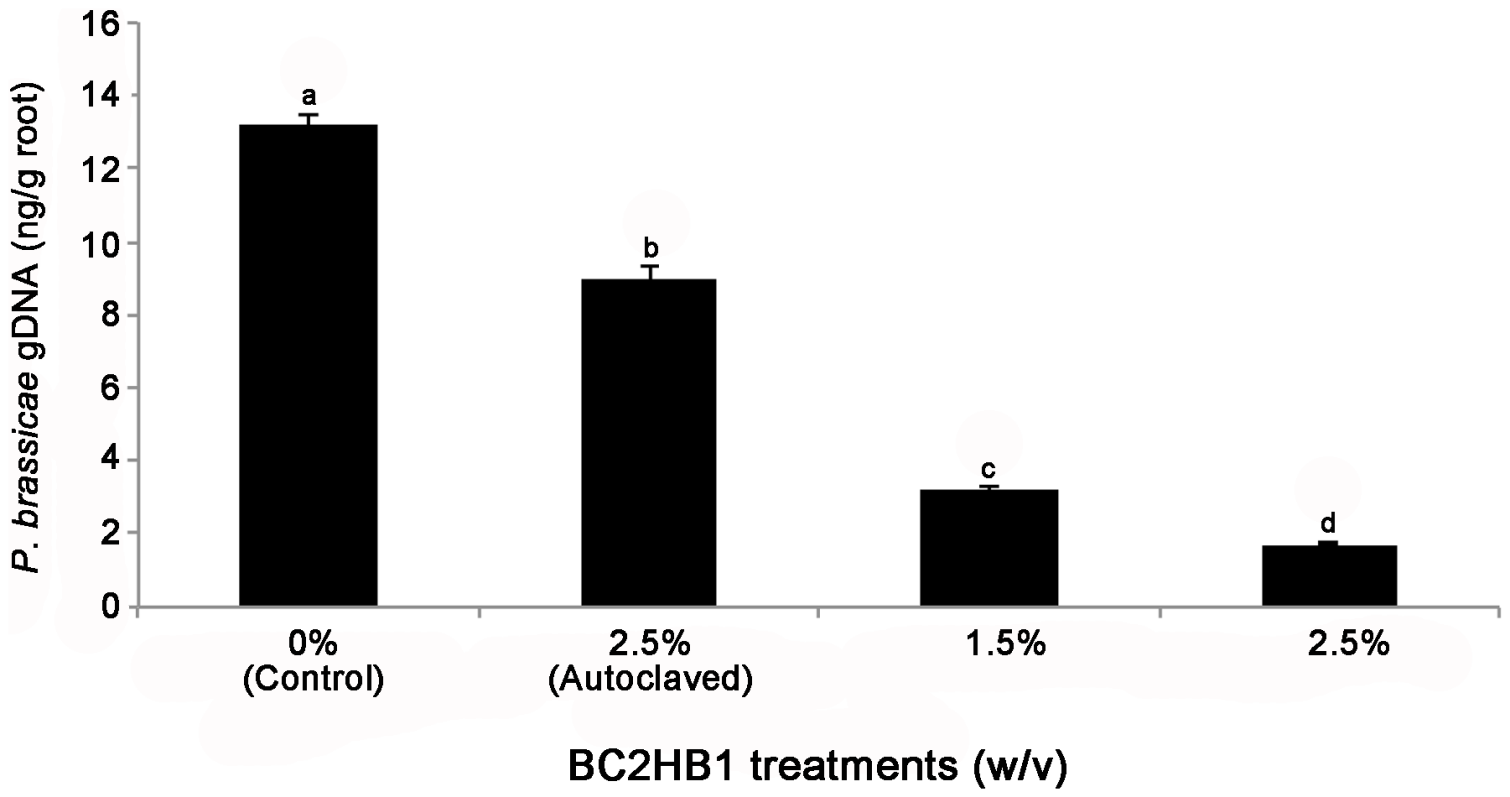
The effect of BC2HB1 treatments on the amount of *Plasmodiophora brassicae* in canola roots (ng DNA/g fresh root, qPCR) at 14 days after seeding. The BC2HB1 formulation was incorporated at 1.25% or 2.5% (w/v) into the planting mix prior to seeding and resting spores of *P. brassicae* were applied at 2×10^5^ spores pot^−1^ after seeding (n = 9).

**Figure 3:**
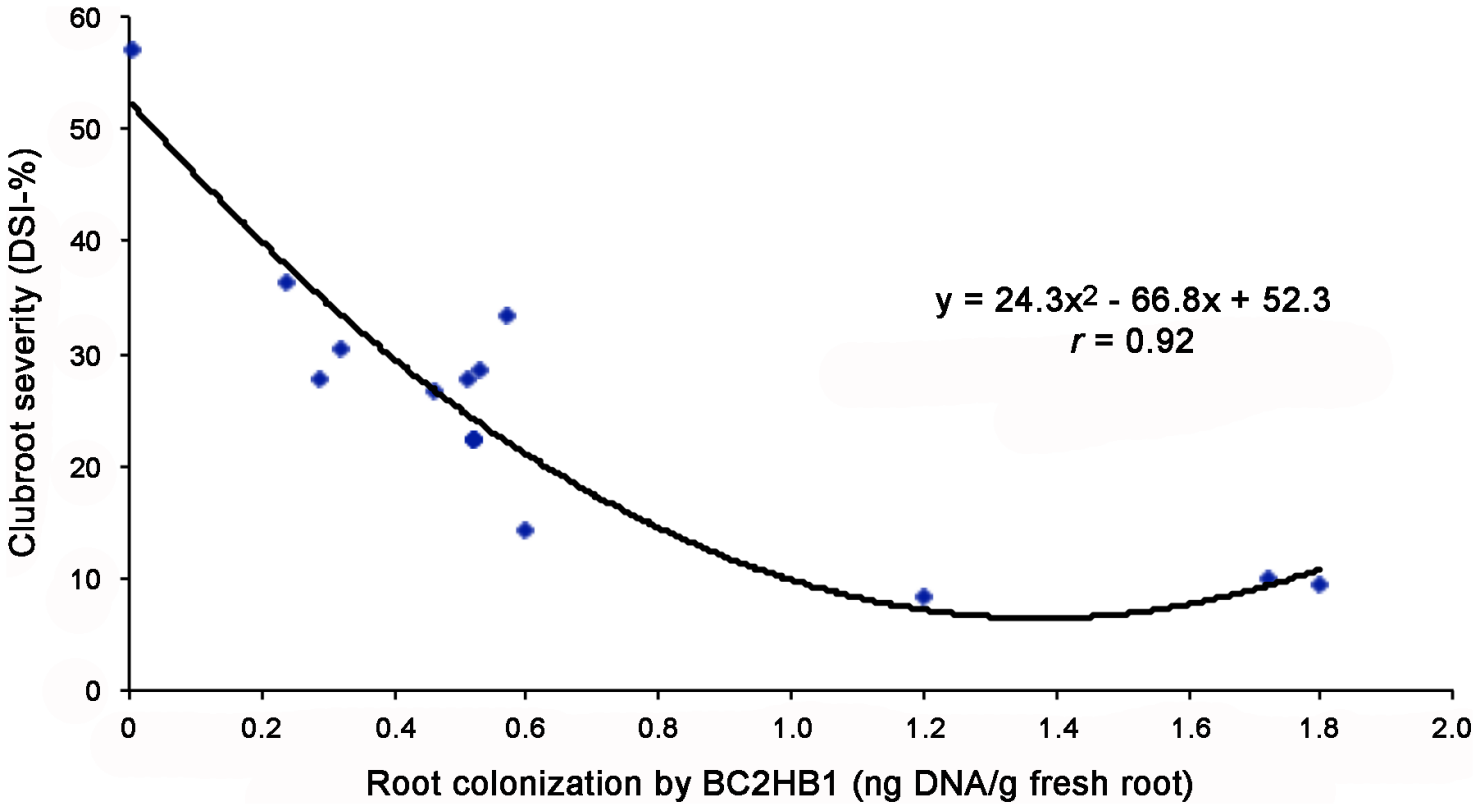
The relationship between colonization of canola roots by BC2HB1 (ng DNA/g of fresh root) at 14 days after seeding and clubroot severity (DSI) at 5 weeks after seeding. The plants were inoculated with *Plasmodiophora brassicae* at 2×10^5^ spores pot^−1^ (low inoculum dose).

The amount of Pb DNA in root samples was generally reduced by Hc treatments (Figure 2), and the higher Hc rate (2.5%) resulted in a more significant reduction in Pb DNA than did the lower rate (1.25%). There was a negative correlation (*r* = 0.92, *P*<0.001) between the amount of Hc DNA detected in roots at 14 DAS and subsequent clubroot severity observed at 5 weeks after seeding (Figure 3) at the lower pathogen inoculum level (2×10^5^ spores pot^−1^) only. No such correlation was observed at the higher dose of pathogen inoculum (2×10^6^ spores pot^−1^) (data not shown).

### Observation of Hc and Pb in canola roots using confocal microscopy

**Figure 4:**
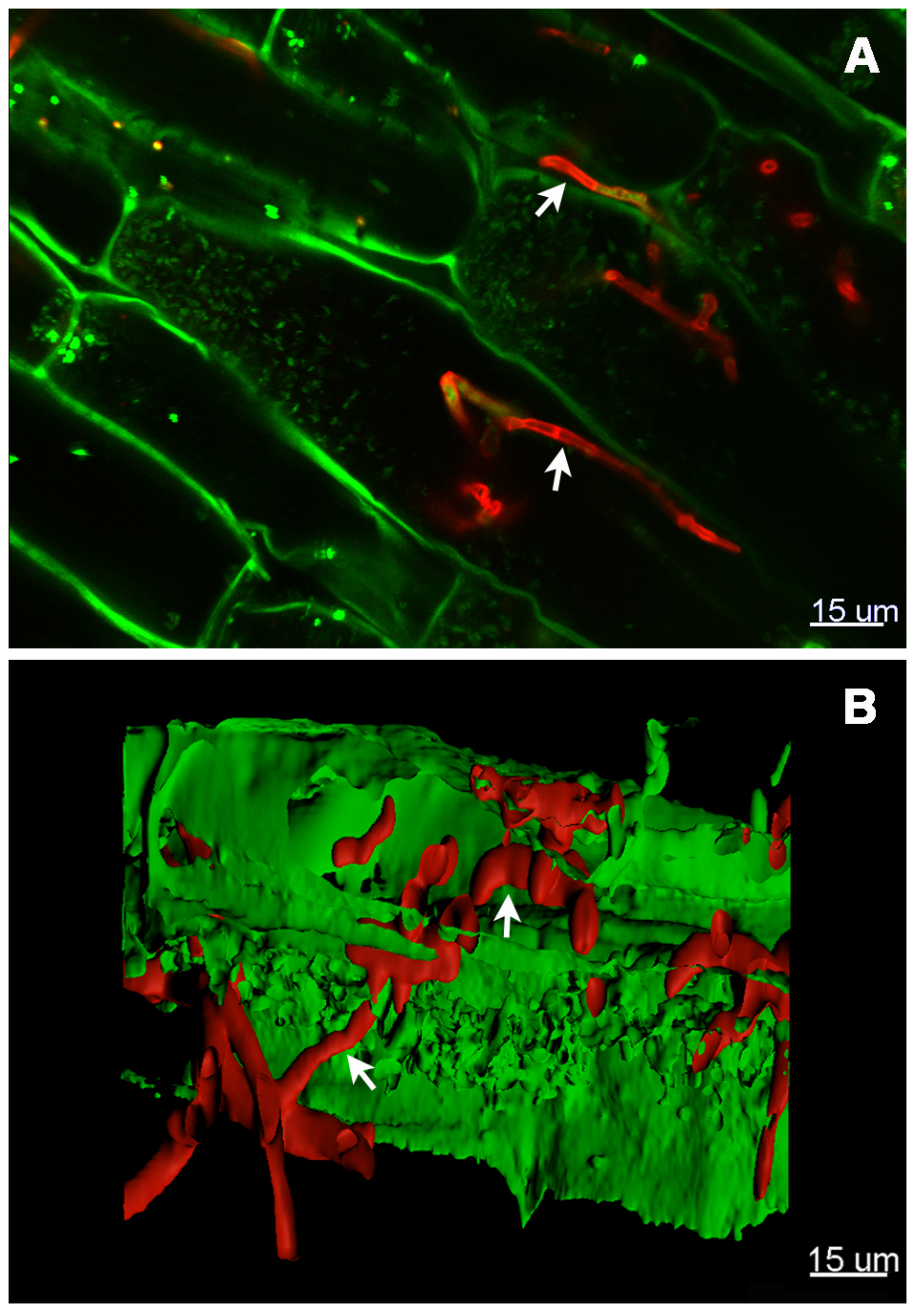
Colonization of canola roots by *Heteroconium chaetospira* BC2HB1 at 14 days after seeding, observed using confocal microscopy. A: fungal hyphae (arrows) in the cortical tissue of a canola root; B: An animation of a profile view of root colonization by fungal hyphae (red, arrow pointed), constructed from 60 confocal microscopic images taken every 1.5 μm from top to bottom of the root sample (in green color).

Colonization of epidermis and cortical tissues by Hc hyphae was observed commonly in canola roots treated with the fungus (Figure 4), but not in any of the control roots (data not shown). The extent of root colonization was not clearly differentiable between the two Hc rates applied. Additionally, the Hc hyphae were not observed in root hairs.

### Activity of phenylalanine ammonia lyases (PAL)

The PAL activity induced by Hc varied depending on the plant tissue assayed and on the presence of Pb inoculum, with generally higher activity in stem tissues and the lowest activity in leaves. When canola plants were treated with both Hc and Pb, PAL activity generally increased in all plant tissues relative to Pb inoculated or non-inoculated controls (Table 3). PAL activity in plants treated with Hc alone was higher only in stems, variable in leaves, but unchanged in roots when compared to that in the controls. The effect of Hc application rate on PAL activity was inconsistent, although there was a slight tendency of higher PAL activity with the lower Hc rate.

**Table 3:** Mean phenylalanine ammonia lyase (PAL) activity in fresh leaves, stems and roots of canola plants treated with *Heteroconium chaetospira* strain BC2HB1 at 1.25 or 2.5% (w/v) with or without inoculation with *Plasmodiophora brassicae* (Pb) (n = 6)^x^

Treatment and rate (w/v)	PAL activity (ηmol trans-cinnamic acid min^−1^g^−1^ tissue)^y^		
	Leaves	Stems	Roots
Negative control (no Pb)	64±2 c^z^	140±21 cd	72±21 d
Pathogen control (Pb)	40±1 e	116±10 d	144±15 c
BC2HB1 (1.25%)	84±3 b	296±15 a	24±4 e
BC2HB1 (2.5%)	50±2 d	248±13 b	148±23 c
BC2HB1 (1.25%) + Pb	96±10 a	232±14 b	252±15 a
BC2HB1 (2.5%) + Pb	84±4 b	156±25 c	216±13 b

x Inoculated with 2×10^5^ spores of *P. brassicae* pot^−1^.

y Assessed at 14 days after treatment.

z Data from repeated trials were pooled for analysis. Means in a column followed by the same letter do not differ (protected LSD at *P* = 0.05).

### Expression of canola (*Bn*) genes in roots induced by Hc and Pb

Based on the transcript levels of nine *Bn* genes encoding enzymes involved in several biosynthetic pathways connecting potentially to plant defence, *BnOPR2* (involved in JA biosynthesis) was upregulated by 6 and 18 fold, respectively, by Pb alone and Hc plus Pb, relative to control (Table 4). The expression of *BnACO* (involved in ET biosynthesis) was increased by Hc plus Pb (24 fold), while that of *BnSAM3* (also in the ET pathway) was not affected, relative to the control. *BnAAO1* (auxin biosynthesis) was upregulated by Pb alone and Hc plus Pb, but the transcription was 3-fold stronger with the later treatment (Table 4). In contrast, genes involved in phenylpropanoid pathways (*BnCCR* and *BnOPCL*) were activated when compared to that of the control but not to that of Pb inoculation.

**Table 4:** The expression (transcript levels) of nine genes potentially related to defence responses in canola at 14 days after a treatment with *Heteroconium chaetospira* BC2HB1 plus *Plasmodiophora brassica* (Pb) or Pb alone, relative to the control (n = 8).

Gene family^x^	Metabolic pathway	Transcript levels relative to control (fold-change)^y^	
		Pb only	BC2HB1 + Pb
*BnSAM3*	Ethylene	0.66±0.15	1.14±0.12
*BnACO*	Ethylene	0.50±0.20	23.97±3.80**^z^
*BnOPR2*	Jasmonic acid	5.75±0.60*^z^	18.09±3.7**
*BnAAO1*	Auxin	12.77±2.5*	38.53±4.75**
*BnPR-1*	PR-1 protein	6.70±1.21*	5.32±1.20*
*BnPR-2*	PR-2 protein	0.11±0.01	6.9±1.08**
*BnPR-5*	PR-5 protein	1.02±0.25	1.02±0.32
*BnCCR*	Phenylpropanoid	1.20±0.30	2.05±0.22*
*BnOPCL*	Phenylpropanoid	1.30±0.25	1.78±0.40*

x Primers used for quantification of the *BnPR-1*, *BnPR-*2 and *BnPR-5* gene families in qPCR were based on Potlakayala et al. (2007), and those for other gene families were based on Zhao et al. (2009).

y Mean transcript levels were normalized using the actin gene as a house-keeping gene (Zhao et al., 2009), averaged over the two repetitions of the test.

z Data from repeated trials were pooled for analysis. Means without an asterisk did not differ from the control. Those with one asterisk (*) were significantly higher than that of control, and those with two asterisks (**) were significantly higher than that in roots inoculated with the pathogen only.

The gene expression related to PR-1 protein biosynthesis (*BnPR-1*) was induced by Pb alone relative to the control, while the *BnPR-2* and *BnPR-5* activity was not affected (Table 4). With the treatment of Hc plus Pb, however, the transcript level of *BnPR-1* was not increased further whereas the expression of *BnPR-2* was upregulated by 60 fold when compared Pb inoculation alone. The genes that encode enzymes involved in the biosynthesis of PR-5 protein or phenylpropanoid were not affected.

## Discussion

Comparison of rDNA sequences indicated that the putative identification of isolate BC2HB1 as *H. chaetospira* is correct; the ITS regions of 5.8s rDNA for BC2HB1 is highly similar (>97%) to those of a *H. chaetospira* isolate deposited previously in the GenBank.

Previous studies had shown that Hc colonized the roots of Chinese cabbage [11], [38], where it suppressed clubroot [8] and Verticillium Yellows [13] through induced resistance [23]. However, the mechanism for this induced resistance was not unknown. In the current study, confocal microscopy and qPCR analysis confirmed the endophytic colonization of canola roots by the isolate BC2HB1 of Hc. This colonization is likely relevant to clubroot suppression as in the previous cases with Chinese cabbage [23], [39]. The high Hc application rate (2.5%) generally resulted in more Hc DNA but lower Pb DNA in canola roots (Table 3, Figure 2) than the low rate (1.5%), and this may contribute to the greater clubroot suppression observed with the high Hc rate. In the current study, Hc was also more effective against lower (2×10^5^ spores) than higher (2×10^6^ spores) Pb inoculum, and this result was consistent with those on Chinese cabbage [40], [41].

Hc treatments generally increased PAL activity, but expression of the *BnCCR* and *BnOPCL* genes was not upregulated. Accumulation of PAL affects the biosynthesis of several secondary metabolites via phenylpropanoid pathways, such as lignins, flavonoids, phytoalexins and antioxidants [42]. Some of these metabolites play an important role in plant defence [43]. PAL is an critical enzyme catalyzing the first step of phenylpropanoid biosynthesis controlled by a multi-gene family [35]. The *OPCL* gene (encoding OPC-8:0 CoA ligase) plays important roles in phenylpropanoid pathways by generating cinnamyl CoA esters of hydroxycinnamic acids [44] that act as intermediates in the biosynthesis of an array of phenolic secondary metabolites [45]. *CCR* encodes the enzyme that catalyzes the conversion of cinnamoyl-CoAs into corresponding cinnamaldehydes, a control point regulating to the overall carbon flux toward lignin production [46]. It appears that neither *OPCL* nor *CCR* was upregulated by Hc plus Pb when compared to Pb inoculation alone. This result supports our initial report that Hc did not increase the expression of *BnCCR* and *BnOPCL* based on microarray analysis [28].

There is little or no information on activation of plant-defence genes associated with ISR mediated by Hc or any other DSE. Most plant-defence genes are regulated by endogenous plant hormones, including the salicylic acid (SA), jasmonic acid (JA) and ethylene (ET) [35]. In the current study, transcript levels for genes encoding enzymes involved in JA and ET biosynthesis were significantly higher in roots treated with Hc plus Pb than with Pb alone. The gene activity related to the biosynthesis of auxin was upregulated by Pb alone, but enhanced a further 3 fold by Hc plus Pb. These results indicate that canola roots respond to the Hc plus Pb treatment with activation of several genes involved in ET, JA and auxin biosynthesis, which may contribute to the induced resistance against clubroot.

Upregulation of genes involved in ET/JA biosynthesis contributes to ISR in canola [36], [37]. JA-pathway signaling has been associated with induction of ISR by arbuscular mycorrhizal fungi [47], [48], which enhances host resistance [49], [50] via stimulation of several plant-defence-related genes [51]. Upregulation of *BnOPR2* by Hc may have a similar effect on ISR. JA is synthesized from α-linolenic acid via four enzymatic steps [52], and the gene *OPR* (12-oxophytodienoate reductase) affects the final step of biosynthesis. ET, on the other hand, is synthesized from methionine via three steps [53] catalysed by S-adenosyl methionine synthase (SAM), aminocyclopropane carboxylate synthase (ACS), and aminocyclopropane carboxylate oxidase (ACO). Upregulation of *BnACO* but not *BnSAM* may suggest that the induction by Hc involves only the enzyme in the final step of biosynthesis. This pattern of gene activation related to ET biosynthesis has also been observed in canola roots treated with *Clonostachys rosea*, where *BnACO* was upregulated at 14 DAS but expression of *BnSAM* was not affected [37]. The strong expression of *BnACO* in the current study also contrasts with the general lack of ET-related gene upregulation in mycorrhiza-mediated ISR, where elevated ET levels may have a negative impact on mycorrhizal development [54], [55].

The strong expression of *BnAAO1* (auxin) associated with Pb inoculation is likely induced by infection [56], with clubbing symptoms associated with auxin accumulation [57]. Interestingly, the transcript level of *BnAAO1* was >3-fold higher in plants treated with Hc plus Pb. It is possible that the gene *BnAAO1* may play a dual role here, involved in pathogenesis and symptom development caused by the pathogen as well as in plant defence. Although this is only a preliminary indication in biocontrol of clubroot, there is an increasing body of evidence showing the potential influence of auxin to transcriptional regulation of plant responses against infection [58], [59], [60] and disease development [61]. Auxin biosynthesis in plants is very complex; several pathways have been postulated, but the biosynthetic process and its regulation remain poorly understood [62]. An indole-3-acetic acid amino synthetase has been reported to activate basal plant immunity in rice [63]. In a canola cultivar partially resistant to *Sclerotinia sclerotiorum*, genes involved in biosynthesis of indole-3-acetic acid and aldehyde oxidase (*AAO1*) were upregulated shortly after inoculation with the pathogen [35]. These results indicate that auxin plays a role in plant defence.

The current study provides evidence that ISR mediated by Hc against clubroot is associated with upregulation of several genes involved in JA, ET and auxin biosynthesis. The activation of these genes seems to be independent of SA biosynthetic pathways. In an earlier microarray study, Hc did not activate any gene involved in SA biosynthesis [28]. There are also other studies suggesting that induced resistance via auxin pathways occurs without biosynthesis of SA [61], [63], and some even believe that auxin and SA pathways may be mutually antagonistic in plant defense, while auxin and JA pathways share more commonalities [60]. In general, JA and ET biosynthesis may be associated with ISR [64], [65], while SA is responsible for SAR, often with the involvement of PRs [48], [66].

Although PRs can be induced via SA, JA and ET pathways, their production is associated most often with SAR via SA biosynthetic pathways [67]. The accumulation of PRs via SA pathways has been reported in canola [27], and an exogenous application of SA to broccoli resulted in upregulation of *PR-1* and *PR-2* genes and a moderate reduction in clubroot severity [68]. The strong expression of *BnPR2* (*β*-1,3-glucanase) in this study, coupled with the evidence above, suggests possible involvement of *PR-2* in Hc-mediated plant defence against clubroot, despite assertions that PRs are not essential for ISR [66]. Regulation of the nine genes in relation to the timing and extent of root colonization by Hc is yet to be established, and root colonization by Hc needs to be better quantified. It is also unclear why ISR was less effective against the high dose of Pb (2×10^6^ spores pot^−1^). Nevertheless, the current study demonstrated that application of Hc at a high rate (2.5%) resulted in colonization of canola roots, reduction in the amount of Pb in the root at the peak of secondary infection by the pathogen (14 DAS), and subsequent reduction in clubroot severity. Further research is required to better relate the Hc application rate to root colonization and clubroot suppression, especially in the environment relevant to canola production under field conditions.

## Conclusion

The current study confirmed the taxonomic designation of BC2HB1 as an isolate of *H. chaetospira* and demonstrated endophytic colonization of canola roots by BC2HB1. BC2HB1 suppressed clubroot on canola through induced resistance via concerted upregulation of genes involved in JA, ET and auxin biosynthesis. The PR-2 protein may also be involved in the plant defense. This is the first report on molecular mechanisms of induced resistance mediated by a non-mycorrhizal endophytic fungus.
